# Complete Genome Sequences of Ezakiella coagulans C0061C1 and Fenollaria massiliensis C0061C2

**DOI:** 10.1128/mra.00444-22

**Published:** 2022-07-05

**Authors:** Michael T. France, Jack Clifford, Shilpa Narina, Lindsay Rutt, Jacques Ravel

**Affiliations:** a Institute for Genome Sciences, University of Maryland School of Medicine, Baltimore, Maryland, USA; b Department of Microbiology and Immunology, University of Maryland School of Medicine, Baltimore, Maryland, USA; University of Rochester School of Medicine and Dentistry

## Abstract

Ezakiella coagulans and Fenollaria massiliensis are two obligate anaerobic bacteria in the family *Peptoniphilaceae* and are both uncommon members of the human vaginal microbiota. We isolated a strain of each bacterium from the same vaginal swab specimen and here report the first complete genome sequences of the two species.

## ANNOUNCEMENT

Ezakiella coagulans and Fenollaria massiliensis are obligate anaerobic bacteria in the family *Peptoniphilaceae* (1, 2). Both species are infrequent members of the human vaginal microbiota (VMB), and, when present, typically represent only a small fraction of the community (3). The VMB is often dominated by single species of *Lactobacillus*, and such communities have been associated with decreased risk for several adverse health outcomes (4). However, both E. coagulans and F. massiliensis are typically not found to be coresident with *Lactobacillus* species and instead cohabit communities that are characterized by a more even collection of other obligate and facultative anaerobes (3), e.g., *Gardnerella*, *Prevotella*, and *Atopobium*. The relationship between vaginal health and E. coagulans and F. massiliensis has yet to be determined. Here, we report the complete genome sequences of these two species.

**FIG 1:**
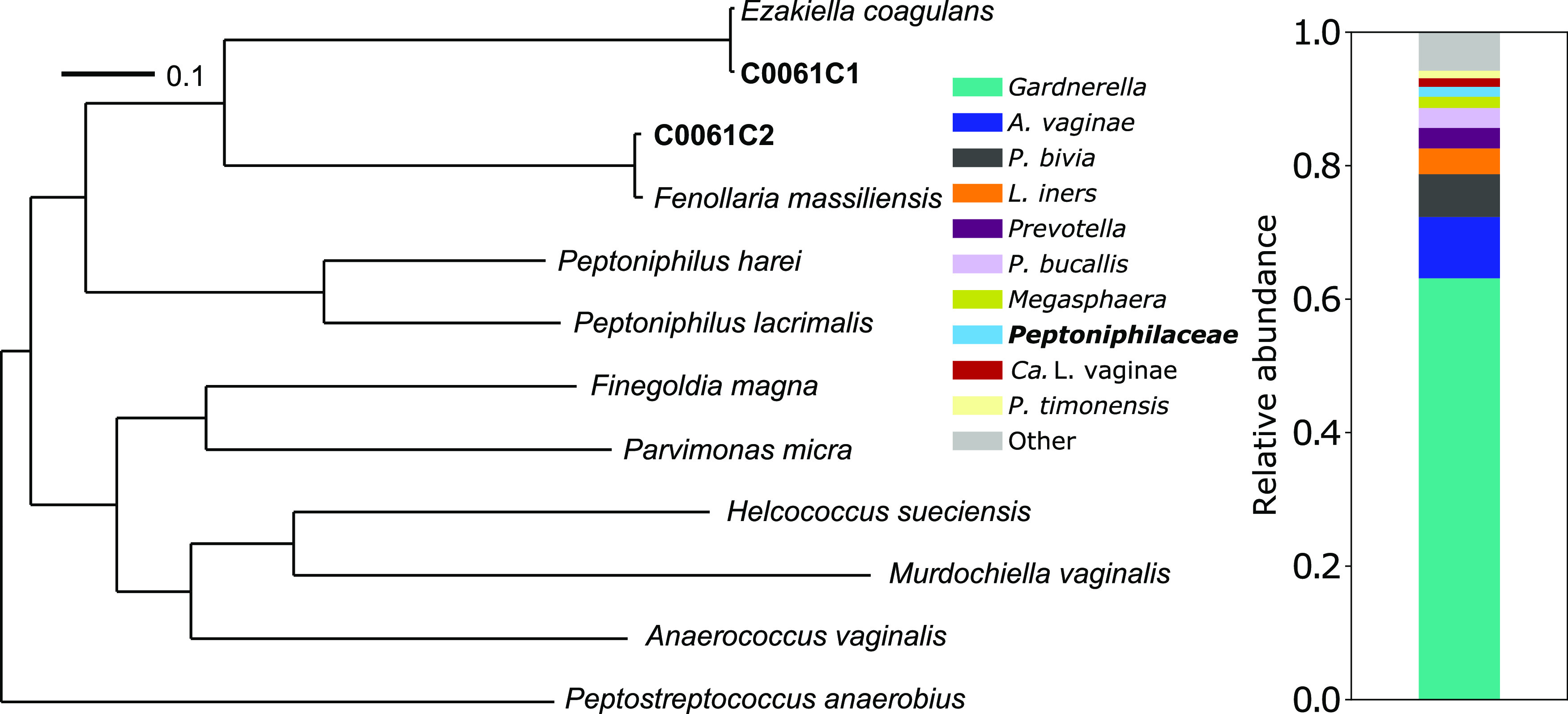
(Right) Taxonomic composition of the VMB at the time of isolation, estimated using previously published shotgun metagenomic data (15) and the VIRGO nonredundant gene catalog (16). (Left) Phylogenetic tree constructed from a concatenated alignment of the amino acid sequences of 514 single-copy core genes. Novel whole-genome sequences of strains C0061C1 and C0061C2 (bold) are most similar to conspecific draft genome sequences. Available genome sequences of E. coagulans (GenBank assembly accession number GCF_003096635.1), F. massiliensis (GenBank assembly accession number GCF_000312505.2), Peptoniphilus harei (GenBank assembly accession number GCF_900638565.1), Peptoniphilus lacrimalis (GenBank assembly accession number GCF_000378725.1), Finegoldia magna (GenBank assembly accession number GCF_013267535.1), Parvimonas micra (GenBank assembly accession number GCF_003454775.1), Helcococcus sueciensis (GenBank assembly accession number GCF_000423145.1), Murdochiella vaginalis (GenBank assembly accession number GCF_900119705.1), Anaerococcus vaginalis (GenBank assembly accession number GCF_016127475.1), and Peptostreptococcus anaerobius (GenBank assembly accession number GCF_900454605.1) were obtained from the NCBI RefSeq database.

One strain each of E. coagulans (C0061C1) and F. massiliensis (C0061C2) were isolated from a midvaginal swab specimen collected from a woman who identified as African American (Fig. 1, right). Swab material was first resuspended in 1 mL of Brucella broth (with hemin and vitamin K) and then plated on human blood Tween bilayer agar (5). The plate was incubated for 72 h under anaerobic conditions (gas mixture: 5% H_2_, 10% CO_2_, and 85% N_2_), and then the strains were isolated. Genomic DNA was extracted, as described previously (5), with the MasterPure complete DNA purification kit (Lucigen, Middleton, WI, USA), sequencing libraries were prepared using the SMRTBell Express template preparation kit 2.0 (Pacific Biosciences [PacBio], Menlo Park, CA, USA), and libraries were size selected targeting 17 kbp using a BluePippin system (Sage Science, Beverly, MA, USA). Sequencing was performed using a Sequel II instrument and single-molecule real-time (SMRT) Cell 8M (PacBio) and produced 2,431,328 reads for C0061C1 (15,387× coverage; read *N*_50_, 12,999 bp) and 2,951,446 reads for C0061C2 (23,111× coverage; read *N*_50_, 12,997 bp). Read correction, trimming, and assembly were performed using Canu (6) (v2.1.1; coverage limit, 1,500×; target genome size, 2 Mbp; minimum read length, 1 kbp). Each assembly produced a single large contig, which was circularized using Simple-Circularise (https://github.com/Kzra/Simple-Circularise) (v1; default settings), rotated using Circlator (7) (v1.5.5; default settings), and taxonomically identified using GTDB-Tk (8) (v1.5.1; default settings).

E. coagulans C0061C1 had a genome that was 1.99 Mbp in length (GC content, 35.2%) and encoded 3 rRNA operons, 49 tRNAs, and 1,904 additional coding sequences. The genome of F. massiliensis C0061C2 had a smaller length of 1.61 Mbp (GC content, 34.7%) and encoded 4 rRNA operons, 48 tRNAs, and 1,514 additional coding sequences. Prediction and annotation of coding sequences were performed using the NCBI Prokaryotic Genome Annotation Pipeline (PGAP) (9–11) (v6.0; default settings). Available whole-genome sequences of other members of *Peptoniphilaceae* and the outgroup Peptostreptococcus anaerobius were acquired from the RefSeq database, and genes present in all strains, including C0061C1 and C0061C2, were identified using orthoMCL (12) (v2.0.9; sequence identity, 70%; E value, ≤10^−5^), aligned using ClustalW2 (13) (v2.1; default settings), and concatenated. A phylogenetic tree (Fig. 1, left) was then constructed from the alignment using RAxML-NG (14) (v1.0.1; molecular evolutionary model, LG; starting trees, 10 parsimony and 10 random [bootstrapping converged after 50 replicates]). E. coagulans C0061C1 and F. massiliensis C0061C2 were most closely related to draft genome sequences from conspecific strains, and the two species were sister to one another.

The swab specimen was collected after informed consent was obtained from the participant, who also provided consent for storage of the material and its use in future research studies related to women’s health. The original study was approved by the University of Maryland School of Medicine institutional review board.

### Data availability.

The E. coagulans and F. massiliensis genome sequences were deposited in NCBI GenBank with the accession numbers CP096650 and CP096649, respectively. The raw PacBio sequence reads were deposited in the NCBI Sequence Read Archive (SRA) under the BioProject accession number PRJNA832302, with the SRA accession numbers SRX15015913 and SRX15015914. All scripts used in the assembly and analysis of the genome sequences are available at https://github.com/ravel-lab/Pepton_WGS.
